# *Botrytis cinerea* differentially induces postharvest antioxidant responses in ‘Braeburn’ and ‘Golden Delicious’ apple fruit

**DOI:** 10.1002/jsfa.9827

**Published:** 2019-07-08

**Authors:** Tuyet TA Bui, Sandra AI Wright, Anders B Falk, Tanja Vanwalleghem, Wendy Van Hemelrijck, Maarten LATM Hertog, Johan Keulemans, Mark W Davey

**Affiliations:** ^1^ Lab. of Fruit Breeding and Biotechnology, Department of Biosystems, Faculty of Bioscience Engineering KU Leuven Leuven Belgium; ^2^ Section of Biology Faculty of Engineering and Sustainable Development, University of Gävle Gävle Sweden; ^3^ Valthornsvagen 38B, SE‐756 50 Uppsala Sweden; ^4^ Department of Mycology, Proefcentrum Fruitteelt vzw Sint‐Truiden Belgium; ^5^ Division of MeBioS, Department of Biosystems, Faculty of Bioscience Engineering KU Leuven Leuven Belgium

**Keywords:** *Malus × domestica*, postharvest storage, *Botrytis cinerea*, antioxidant metabolism

## Abstract

**BACKGROUND:**

The fruit of two apple cultivars – ‘Braeburn’, which is susceptible to inoculation with *Botrytis cinerea*, and the less susceptible cv. ‘Golden Delicious’ – were investigated with respect to their response to inoculation with *B. cinerea*. Successful infection by *B. cinerea* leads to an oxidative burst and perturbation of plant redox homeostasis. To investigate the interaction between apple fruit and *B. cinerea*, antioxidant metabolism in fruit samples from sun‐exposed and shaded sides of different tissue types was measured over time.

**RESULTS:**

The sun‐exposed tissue of ‘Braeburn’ had higher initial levels of total vitamin C in the peel and phenolic compounds in the flesh than ‘Golden Delicious’, despite its greater susceptibility to gray mold. A substantial antioxidant response was recorded in diseased ‘Braeburn’ fruit 14 days after inoculation, which involved an elevated superoxide dismutase activity and ascorbate peroxidase activity, a progressive oxidation of total vitamin C, and a decrease in peroxidase activity and phenolic content. Disease development was slower on the sun‐exposed sides than on the shaded sides.

**CONCLUSION:**

The two cultivars appeared to utilize different strategies to defend themselves against *B. cinerea*. ‘Golden Delicious’ almost entirely escaped infection. Preharvest exposure of apple fruit to high light / temperature stress appears to prepare them to better resist subsequent postharvest attack and disease. © 2019 The Authors. Journal of The Science of Food and Agriculture published by John Wiley & Sons Ltd on behalf of Society of Chemical Industry.

## INTRODUCTION

Apples (*Malus × domestica* Borkh.) are a major source of antioxidants, such as ascorbic acid (AsA), also known as vitamin C, and phenolic compounds.^1^ Preharvest conditions during the cultivation of apples have an impact on the levels of phytochemicals and these can relate to the postharvest resistance of apple fruit to pathogens. *Botrytis cinerea* is a necrotrophic pathogen, which causes preharvest and postharvest disease (gray mold) in apple.^2^, ^3^

Stress conditions during the postharvest period can lead to the accumulation of reactive oxygen species (ROS), such as superoxide (O_2_^−^), singlet oxygen (^1^O_2_) hydroxyl radicals (OH^−^), and hydrogen peroxide (H_2_O_2_), which can have a detrimental effect on cellular metabolism.^4^ However, plant cells are to some extent protected from ROS by their antioxidant system, consisting of radical scavengers and detoxifying agents.^5^ The first redox reaction is catalyzed by the enzyme superoxide dismutase, which converts superoxide (O_2_^−^) into H_2_O_2_. An excess of H_2_O_2_ is toxic to cells, so H_2_O_2_ needs to be metabolized further. Flavonoid peroxidases (POX; EC 1.11.1.7), ascorbate peroxidase (APX; EC 1.11.1.11), and catalase (CAT; EC 1.11.1.6) all have the ability to convert H_2_O_2_ to water. Flavonoid peroxidases detoxify H_2_O_2_ by using flavonoids as substrates. Catalase is thought to metabolize H_2_O_2_ to water and oxygen via the iron‐heme groups that are attached to the enzyme. Ascorbate peroxidase detoxifies H_2_O_2_ by using AsA as a reducing agent, yielding monodehydroascorbate (MDHA), a radical form, and dehydroascorbate (DHA) – both oxidized and reduced back to AsA by monodehydroascorbate reductase (MDHAR) and dehydroascorbate reductase (DHAR), respectively. The chemical balance between the reduced and oxidized forms of ascorbic acid is maintained through the AsA‐glutathione cycle.^6^ Maintaining a large pool of AsA and an efficient recycling mechanism is crucial for the protection of cells against oxidative stress. Dehydroascorbate and MDHA are transient species, and these oxidized forms thus typically constitute minor proportions of the total vitamin C content in plant tissues.^7^ It is generally accepted that preharvest exposure and adaptation to stress, for instance via high light / high‐temperature regimes, increase the antioxidant content in apples.^8^, ^9^ Apple tissues with high levels of antioxidants, and in particular of AsA, are less susceptible to inoculation with *B. cinerea*.^10^

In the early stages of gray mold infection, H_2_O_2_ accumulates and is converted to ROS, resulting in host cell death.^11^ The plant‐generated oxidative burst is induced and utilized by *B. cinerea* to colonize the plant successfully.^11^, ^12^ The functions of ROS in plants and *Botrytis* are multiple, including signaling aspects.^13^, ^14^ During pathogenesis, there is histochemical evidence that *B. cinerea* produces O_2_^.‐^ and H_2_O_2_ in hyphal germ tubes at the infection site,^15^ and that its superoxide dismutases (SOD; EC 1.15.1.1) may be involved in pathogenicity.^16^

Two apple cultivars, ‘Braeburn’ and ‘Golden Delicious’ were selected to elucidate further the influence of preharvest sun exposure and antioxidant metabolism on subsequent postharvest disease development caused by *B. cinerea*. The relationships between the antioxidant content and antioxidant enzyme activity in both peel and flesh were analyzed in relation to the susceptibility of fruit to *B. cinerea*, studying both the sun‐exposed and shaded sides of apple fruit.

## MATERIALS AND METHODS

### Fruit

The ‘Braeburn’ (‘Br’) and ‘Golden Delicious’ (‘GD’) apple cultivars were selected based on their differences in color and susceptibility to *B. cinerea*. ‘Br’ has a bicolored fruit with a clear difference between the red, sun‐exposed and the green, shaded side of the fruit, whereas both sides of the ‘GD’ fruit are different shades of yellow. Random samples of healthy apple fruit of comparable size were harvested in the fall of 2008 (22 September 2008 for ‘GD’ and 22 October 2008 for ‘Br’) from trees grown at the Research Station for Fruit Cultivation (Proefcentrum Fruitteelt), Sint‐Truiden, Belgium (50° 46′ 21.82″ N, 5° 09′ 36.10″ E). Apple fruit were stored at Proefcentrum Fruitteelt under controlled atmosphere conditions (for ‘Br’ 0.5 °C, 1–2% O_2_, 2–2.5% CO_2_, 95% RH, whereas for ‘GD’ 1 °C, 2.5–3.0% O_2_, < 8% CO_2_, 95% RH), until July 2009, when inoculation was carried out at KU Leuven, Belgium.

### Sampling for biochemical analyses

For the analysis of antioxidant metabolism and of antioxidant enzyme activity, samples of apple tissue taken from the center of the point of inoculation were excised, using a 0.5 cm diameter cork‐borer, weighed, and immediately frozen in liquid nitrogen. The upper 0.2 cm of the fruit plug was considered peel sample, and the remainder, at 0.2 to 2.0 cm from the surface, was considered flesh (hypanthium – fruit cortex) sample.

### Inoculation with *B. cinerea*

*Botrytis cinerea* strain B05.10 was obtained from the Center of Microbial and Plant Genetics (CMPG unit PFI) at KU Leuven. Cultivation and spore harvesting were performed as described by Broekaert *et al*.^17^

Round apple wounds, 6 mm deep and 3 mm wide, were created by pressing a sterile metal tool into the equators of each apple cheek. The apples were treated on both the sun‐exposed and the shaded sides of the fruit by introducing either 10 μL of sterile water (mock treatment) or 10 μL of spore suspension (inoculated treatment) into each wound. The spore suspension contained *B. cinerea* spores at a concentration of 1.5 × 10^5^ mL^−1^. Controls were neither wounded nor inoculated. Following treatment, fruit were stored at room temperature (20 ± 2 °C), at 100% RH for the first 24 h, and then at 80% RH for the remainder of the experiment by placing them in sealed plastic bags. At the start of the experiment, 46 similar fruit per cultivar were selected. Ten fruit per cultivar were immediately employed as non‐inoculated control fruit at 0 days. Of the 36 remaining fruit per cultivar, 12 were designated as non‐inoculated controls for 5 days and 14 days post inoculation. Similarly, 12 fruit were mock‐inoculated and 12 were inoculated with *B. cinerea*. For each treatment, six fruit per cultivar were evaluated 5 days after treatment and the remaining six fruit per treatment 14 days after treatment. When scoring the infection response, the inoculated fruit were classified either as tolerant, apples without disease symptoms, or as susceptible, apples with symptoms.

### Evaluation of disease progress

The quantification of disease symptom development was carried out by measuring the diameter of the lesion (in mm) emerging from the point of inoculation in the peel. Three treatments (control, mock‐inoculated, and *B. cinerea*‐inoculated) were scored on both the sun‐exposed and shaded sides of the two apple cultivars at the three time points of the experiment: 0 days, 5 days and 14 days post inoculation. The number of apples with characteristic symptoms of gray mold were counted 5 and 14 days post inoculation.

### Biochemical measurements

#### Apple tissue extraction

Extraction of fruit tissue was carried out according to Ahn *et al*.^18^ Protein content was determined according to Bradford,^19^ using bovine serum albumin as a standard.

#### Analysis of antioxidant enzyme activity

All enzymatic kinetic analyses were implemented on quartz 96‐well microtiter plates (Hellma™, VWR, Belgium) in a total assay volume of 200 µL, consisting of 20 μL of enzyme extract and 180 μL of reaction mixture. Readings were recorded every 30 s using a Multiskan spectrum‐microplate spectrophotometer (Thermo Lab Systems, Helsinki, Finland). Enzyme activity was expressed as units per g of fresh weight.

The activity of SOD was assayed following Banowetz *et al*.^20^ The commercial standard of superoxide dismutase from bovine liver (15 000 units) was purchased from Sigma‐Aldrich (Saint Louis, MO, USA). A stock solution of the SOD standard was prepared of 2 g L^−1^ in water, equivalent to 3277 units per mg protein, and stored at −20 °C. A standard curve was generated for the range of 1 to 100 U mL^−1^.

The activity of POX was analyzed essentially as described by Chance and Maehly,^21^ with slight modifications. First, 180 μL of the reaction mixture, containing 50 mmol L^–1^ potassium phosphate buffer pH 7.0, 0.01 mol L^–1^ EDTA (ethylene‐diamine‐tetra‐acetic acid), 0.02 mol L^–1^ pyrogallol and 1.47 mmol L^–1^ H_2_O_2_, was added to the wells of a 96‐well plate. Subsequently, 20 μL of enzyme extract was added to the wells, with triplicates for each treatment replicate. Finally, the plate was agitated briefly and incubated in the microplate spectrophotometer for 30 s before absorbance was recorded at 470 nm.

The activity of APX was monitored by using a slightly modified version of Nakano and Asada's method.^22^ First, 180 μL of the reaction mixture, containing 50 mmol L^–1^ potassium phosphate buffer pH 7.0, 0.1 mmol L^–1^ EDTA, 0.88 mmol L^–1^ AsA, and 0.1 mmol L^–1^ H_2_O_2_, was added to the wells of a 96‐well plate. Subsequently, 20 μL enzyme extract was added to the wells, using triplicates for each sample. Finally, the plate was agitated slightly and incubated in the microplate spectrophotometer for 30 s, after which absorbance was recorded at 290 nm (extinction coefficient of 2.8 mM ^−1^ cm^−1^). The reaction rate was measured during the first 30–90 s.

The activity of CAT was measured based on Aebi's method,^23^ with slight modifications. The reaction mixture contained 10 mmol L^–1^ H_2_O_2_ in 50 mM potassium phosphate buffer at pH 7.0. First, 180 μL of reaction mixture was distributed into each well of a quartz 96‐well microtiter plate (VWR), then 20 μL of CAT standard at eight different concentrations, was added in triplicate for each concentration of CAT standard, in three columns of the 96‐well plate. Then, 20 μL enzyme extract was added to separate wells in another part of the plate, with triplicates for each treatment replicate. Finally, the plate was agitated briefly and incubated in the microplate spectrophotometer for 30 s before the disappearance of hydrogen peroxide was monitored at 240 nm. The rate was calculated from the initial linear portion of the curve. A standard of CAT from bovine liver was purchased from Sigma‐Aldrich (Steinheim, Germany).

#### Analysis of antioxidants

The phenolic content in apple fruit tissues was measured according to a modified version of the Folin–Ciocalteu assay.^24^ Total vitamin C was extracted by transferring approximately 0.2 g of tissue to a 1.5 mL reaction tube followed by addition of 1 mL of extraction buffer containing 3% metaphosphoric acid and 1 mmol L^–1^ EDTA. The tubes were vortexed and stored on ice until centrifugation for 10 min at 10 000×*g* in a precooled microcentrifuge. The supernatant was filtered through a PVDP filter with 0.45 µm pore size (Millipore, Brussels, Belgium) and analyzed immediately for total vitamin C content, consisting of AsA and DHA. Total vitamin C content was quantified by high‐performance liquid chromatography (HPLC) analysis, as described by Franck *et al*.,^25^ and expressed on a fresh weight basis.

#### Tissues used in the analysis

Antioxidant enzyme activity and antioxidant content were measured in both the peel and flesh tissues in the three treatments at the three time points of the experiment. An exception was the measurement of phenolic content, for which only flesh tissue was used. The peel tissue could not be analyzed for phenolic compounds, due to the limited amount of tissue available.

### Statistical analysis

Statistical analyses were carried out using R (version 3.5.1, R development core team, Vienna, Austria, 2018). The response variables were continuous, so the errors were assumed to follow a Gaussian distribution. Overall statistical analysis was by multivariate analysis of variance (MANOVA) using the *manova* function in R. Pairwise tests for mean differences were carried out with the *emmeans* function of the emmeans (previously lsmeans) package in R, followed by Tukey's test to control the family‐wise error rate at a predetermined *α* = 0.05. Alternatively, after a significant ANOVA, pairwise t‐tests were performed using the *pairwise.t.test* function in package stats in R, with non‐pooled standard deviation and no correction for multiple comparisons. Both procedures were considered suitable for unbalanced designs. Letters for indicating significant differences among multiple comparisons were generated using the *CLD* function (package emmeans), or the *multcompLetters* function of the multcompView package.

## RESULTS

### Overall statistical analysis

The focus of this study was to examine the effect of treatment on a set of dependent components, in particular, on lesion size, which was analyzed by MANOVA (Table 1). The treatment factor consisted of three levels: *B. cinerea* inoculation, mock inoculation, and control. Other categorical factors included in the MANOVA were: cultivar (levels ‘Br’ and ‘GD’), side (levels sun‐exposed and shaded), tissue (levels peel and flesh), and day (levels 0 days, 5 days and 14 days). Dependent components were lesion size, AsA, phenolics, and enzyme activities (SOD, POX, APX, and CAT). All categorical factors were significant in the overall MANOVA. However, many significant interaction factors were also observed, calling for further analysis.

**Table 1:** Summary of MANOVA for components tested. The dataset was analyzed in two separate MANOVAs with the categorical variables (source of variation) as independent variables (predictors) and the continuous variables (components) as dependent variables

	Source of variation					Interactions									
	Treatment (A)	Cultivar (B)	Side (C)	Tissue^a^ (D)	Day (E)	AB	AC	AD	AE	BC	BD	BE	CD	CE	DE
df^b^	2	1	1	1	2	2	2	2	2	1	1	2	1	2	2
Overall MANOVA	***	***	***	***	***	***	NS	***	***	***	***	***	***	NS	***
Components^b^															
Lesion size	***	***	**	NA	***	***	*	NA	***	NS	NA	***	NA	*	NA
Vit C	***	***	***	***	*	***	NS	*	NS	***	***	NS	***	NS	***
Phe	NS	NS	NS	NA	***	*	NS	NA	NS	NS	NA	NS	NA	*	NA
SOD	***	***	NS	***	*	**	NS	***	***	NS	NS	NS	NS	NS	***
POX	*	***	NS	***	**	NS	NS	NS	NS	NS	NS	NS	NS	NS	***
APX	***	NS	NS	***	NS	NS	NS	***	NS	NS	NS	NS	NS	NS	*
CAT	NS	NS	NS	***	***	NS	NS	NS	NS	NS	NS	NS	NS	NS	***

The summary table is based on the MANOVA in which tissue is included as predictor and phenolic content was excluded, because phenolic data was only available for flesh tissue (Table S1a). The second MANOVA included phenolics and excluded tissue (Table S1b); from which the results for the analysis of phenolics were transferred into the summary table. The analysis was done in R using the MANOVA function of package stats.

a Tissue is not a relevant predictor for the dependent variable lesion size, because peel and flesh have the same lesion size (thus, indicated NA).

b Abbreviations: df, degrees of freedom; Vit C, total vitamin C content; Phe, phenolic content; SOD, superoxide dismutase activity; POX, flavonoid peroxidase activity; APX, ascorbate peroxidase activity; CAT, catalase activity; NS, not significant (*P* > 0.05); NA, not applicable.

* *P* < 0.05.

** *P* < 0.01.

*** *P* < 0.001.

### Development of disease

**Figure 1:**
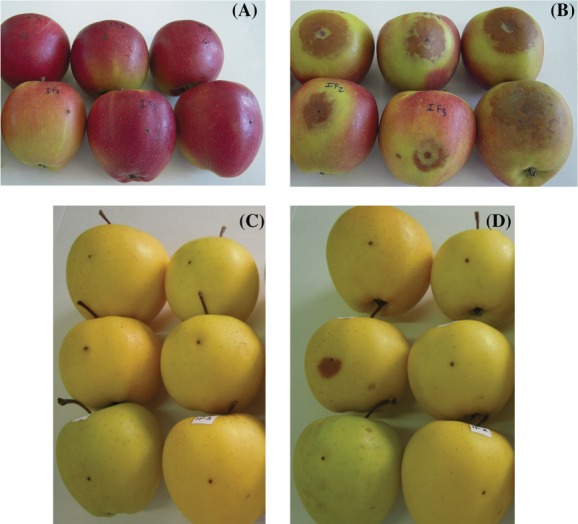
Symptoms of *B. cinerea* at 5 days post inoculation; representative fruit of ‘Braeburn’ sun‐exposed sides (A), and shaded sides (B), and fruit of ‘Golden Delicious’ showing the absence of symptoms on the sun‐exposed sides (C), and occasional symptoms on shaded sides (D).

**Figure 2:**
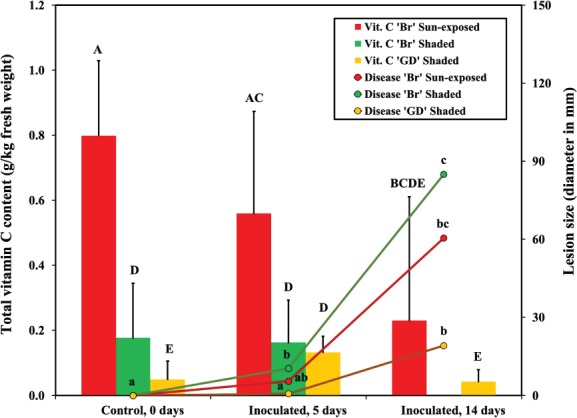
The change over time of total vitamin C content in peel (bars) and in lesion sizes (lines) of apples of the cultivars ‘Braeburn’ (‘Br’) and ‘Golden Delicious’ (‘GD’), at 5 and 14 days after inoculation with *B. cinerea*. The inoculated, sun‐exposed sides of ‘GD’ were symptomless at all time points; thus, the data is not presented. The treatments were: control (neither wounded, nor inoculated) at 0 days post inoculation, and *B. cinerea*‐inoculated (wounded and inoculated with 10 μL of a 1.5 × 10^5^ mL^−1^ *B. cinerea* spore suspension). The results from separate t‐tests of total vitamin C content and lesion size without pooled standard deviations are shown as capital letters (total vitamin C content) and as small letters (lesion size), respectively. Measurements with the same letter were not significantly different at *P* ≤ 0.05.

The MANOVA demonstrated that treatment, cultivar, and day significantly influenced lesion size (Table 1). The factor ‘side’ was also significantly different. The lesion sizes appeared smaller on the sun‐exposed sides than on the shaded sides (Fig. 1). The shaded sides of ‘Br’ fruit were almost all displaying symptoms at 5 days, whereas a similar situation did not appear until 14 days for the sun‐exposed sides (data not shown). At 5 days post inoculation, disease symptoms were observed on both sides of ‘Br’ apple fruit inoculated with *B. cinerea* (Fig. 1(A), (B)). The lesions were tiny on the sun‐exposed sides (Fig. 1(A)), but they had grown significantly in size by 14 days post inoculation (Fig. 2). For ‘GD’ apples, disease symptoms were observed only on the shaded sides of a few ‘GD’ apple fruit (Fig. 1(D)). The sun‐exposed sides of ‘GD’ apples did not develop disease (Fig. 1(C)), even at 14 days. For ‘Br’ shaded sides, the high incidence recorded at 5 days remained for 14 days, indicating early onset of disease. However, for both ‘Br’ sun‐exposed and ‘GD’ shaded fruit, the low disease incidence doubled between 5 days and 14 days (data not shown), indicating a slow progression over time. The ranking of susceptibility for the four tested tissues was identical, regardless of whether disease incidence (percentage infected fruit) or severity (lesion size) was used as measure: ‘Br’ shaded (most susceptible) > ‘Br’ sun exposed > ‘GD’ shaded > ‘GD’ sun exposed (least susceptible). The Spearman rank correlation coefficient between disease incidence and lesion size was equal to 1. In conclusion, ‘Br’ was more susceptible to gray mold than ‘GD’.

### Antioxidant metabolite content

#### Total vitamin C content

The MANOVA demonstrated that all factors significantly influenced total vitamin C content (Table 1). Total vitamin C content tended to be higher in peel as compared to flesh tissues, for both ‘Br’ and ‘GD’ (Table S2). The total vitamin C content of sun‐exposed peel tissue of ‘Br’ was approximately tenfold higher than that of sun‐exposed flesh tissues. Sun‐exposed peel of ‘Br’ had significantly higher initial total vitamin C content than any other tissue examined (Fig. 2 and Table S2); as much as fivefold or sixfold higher than sun‐exposed peel tissue of ‘GD’. Over the course of the experiment, the total vitamin C content in the peel tissues of *B. cinerea*‐inoculated ‘Br’ apples was reduced (Fig. 2). At 14 days post inoculation, when rot of gray mold was extensive on the shaded side of inoculated ‘Br’ fruit, the total vitamin C content in peel was no longer detectable, whereas it was significantly reduced in sun‐exposed apple peel (Fig. 2, and Table S3).

#### Disease development and vitamin C content

The relationship between total vitamin C content and lesion size over time is presented in Fig. 2. Overall, the total vitamin C content in the apples decreased as the lesion size increased. However, the total vitamin C content at 0 days was significantly different among different apple sides and cultivars (Fig. 2). At 5 days post inoculation, the total vitamin C content of the sun‐exposed side of ‘Br’ was still significantly higher than those of the shaded sides of ‘Br’ and ‘GD’, The largest increase in lesion sizes from 5 to 14 days post inoculation was seen on the shaded side of ‘Br’ (Fig. 2).

#### Total phenolic content

In the MANOVA (Table 1), a significant effect on phenolic content was found only for factor day. At the start of the experiment, the phenolic content was significantly higher in flesh tissue of ‘Br’ as compared to the flesh tissue of ‘GD’, especially on the sun‐exposed side (Table S2). In general, sun exposure did not have a significant influence on phenolic content, as seen in samples taken at 0 days (Table S2), and as also indicated by the MANOVA results. There was no difference in phenolic content in ‘Br’ flesh tissues at 14 days when the content in *B. cinerea‐*inoculated treatments was compared by t‐tests to that of the mock‐inoculated and the non‐inoculated controls. However, by dividing the *B. cinerea‐*inoculated ‘Br’ apples at 14 days into susceptible and tolerant reactions, it was apparent that the phenolic content was lower at 14 days only in the susceptible flesh tissue (0.67 ± 0.16 mg kg^−1^), as compared to the control at day 0 (0.92 ± 0.10 mg kg^−1^), or to the controls at day 5 (non‐inoculated control, 1.03 ± 0.13 mg kg^−1^; mock‐inoculated control 1.07 ± 0.15 mg kg^−1^). The phenolic content also decreased in susceptible ‘Br’ flesh from 5 days (0.98 ± 0.20 mg kg^−1^) to 14 days post inoculation.

### Antioxidant enzyme activity

#### Superoxide dismutase (SOD) activity

The MANOVA demonstrated that the factors treatment, cultivar, tissue and day significantly influenced SOD activity (Table 1). A significant increase in SOD activity in ‘Br’ was recorded 14 days post inoculation in *B. cinerea*‐inoculated peel tissue, when compared to any other treatment on any day (Table S3, Table S4a), which, in fact, reflected the response in susceptible apples at 14 days post inoculation (Table S4b). When susceptible apples were examined further at 14 days post inoculation, the peels displayed significantly higher SOD activity on the sun‐exposed sides as compared to the shaded sides (data not shown). Control or mock‐inoculated treatments showed no significant differences over time in SOD activity in peel tissue for either cultivar (Table S3). When the cultivars were compared, e.g. at 0 days, SOD activity was significantly ∼ sixfold higher in flesh of ‘Br’ as compared to flesh of ‘GD’ (Table S2).

#### Flavonoid peroxidase (POX) activity

The MANOVA demonstrated that the factors cultivar, tissue, day, and treatment significantly influenced POX activity (Table 1). The differences in POX activity between cultivars at 0 days are presented in Table S2. At that time, flesh tissue of ‘Br’ had significantly higher POX activity than that of ‘GD’. Flesh tissue generally had higher POX activity than peel tissue for both cultivars (Table S2). At 14 days after inoculation, ‘GD’ did not display any difference in POX activity for control and inoculated treatments, whereas for ‘Br’, POX activity was significantly reduced in the *B. cinerea*‐inoculated (tolerant and susceptible) tissue, flesh and peel combined, when compared to the control or mock‐inoculated tissue (Table S4a). Furthermore, only susceptible apples, and not tolerant ones, had lowered POX activity (Table S4b). Specifically, this lowered POX activity was located in flesh tissues. The POX activity in the peel tissue of ‘Br’ did not change significantly over time (data not shown).

#### Ascorbate peroxidase (APX) activity

The MANOVA demonstrated that the factors tissue and treatment significantly influenced APX activity (Table 1). At 0 days, APX activity was similar in both tissues for both cultivars, and across the sun‐exposed and shaded tissues (Table S2). Significant differences were, however, found at 14 days post inoculation in ‘Br’. At that time, APX activity had increased in the inoculated treatment, as compared to the control (Table S4a). This increase was due to an increase in APX activity in susceptible fruit 14 days post inoculation (Table S4b).

#### Catalase (CAT) activity

The MANOVA demonstrated that the factors tissue and day significantly influenced CAT activity (Table 1). The two apple cultivars displayed similar levels of CAT activity. CAT activity was also similar for sun‐exposed and shaded sides of fruit tissues. Similar CAT activity was observed among the three treatments (Table 1, S1a and S1b). CAT activity was slightly different in peel and flesh of ‘Br’ (Table S2). In flesh tissue, it was significantly higher than in peel tissues on the shaded side (Table S2). CAT activity changed over time (Table 1), increasing steadily in all treatments (data not shown). At 14 days post inoculation, CAT activity was not significantly different between the *B. cinerea*‐inoculated treatment and the non‐inoculated control (Table S4a). The CAT activity of susceptible apples at 14 days post inoculation did not differ from that of the control apples (Table S4b).

#### Overall antioxidant enzyme activity

**Figure 3:**
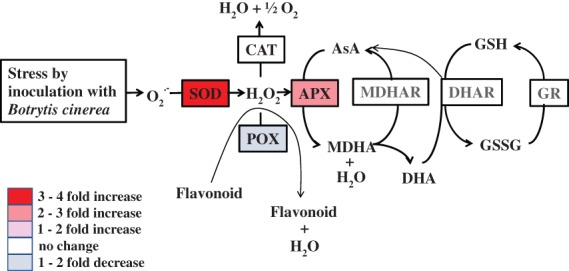
Summary of the antioxidant enzyme responses in ‘Braeburn’ (‘Br’) apple fruit as a consequence of infection by *B. cinerea*. The antioxidant enzyme activity is collectively analyzed for shaded and sun‐exposed tissue, as for peel and flesh tissue. The colors represent the changes in antioxidant enzyme activity of susceptible fruit at 14 days post inoculation, compared to noninoculated control fruit at the same point in time. The changes in enzyme activity were analyzed by ANOVA, followed by pairwise t‐tests. Abbreviations: APX, ascorbate peroxidase; AsA, vitamin C (l‐ascorbic acid); CAT, catalase; DHA, dehydroascorbate; DHAR, dehydroascorbate reductase; GR, glutathione reductase; GSH, glutathione; GSSG, oxidized glutathione; H_2_O_2_, hydrogen peroxide; MDHA, monodehydroascorbate; MDHAR, monodehydroascorbate reductase; O2.‐, superoxide anion; POX, flavonoid peroxidase; SOD, superoxide dismutase.

In the MANOVA (Table 1), the factor treatment strongly influenced SOD and APX activities, and also POX activity, whereas there was no significant effect on CAT activity. The antioxidant enzyme activities of inoculated ‘Br’ at 14 days post inoculation (Table S4a) were analyzed further by pairwise t‐tests, separately comparing the activity in tolerant and susceptible apples with that of control apples at 14 days (Table S4b). It was the susceptible ‘Br’ apples that displayed the observed increase in SOD and APX activity and the decrease in POX activity that had been observed in inoculated apples. The flow chart presented in Fig. 3 summarizes the significant changes reported in Table S4b. The antioxidant enzyme activity in peel and flesh tissues (both sun exposed and shaded sides) of ‘Br’ fruit susceptible to inoculation with *B. cinerea* at 14 days post inoculation are compared with the antioxidant enzyme activity of control fruit at 14 days post inoculation. The colors demonstrate the change in enzyme activity and are based on the analysis presented in Table S4b. Following inoculation of apples with *B. cinerea*, SOD activity increased by 4.1‐fold, APX activity increased by 2.8‐fold, and POX activity decreased by 2.0‐fold.

## DISCUSSION

In the present study, two apple cultivars, ‘Golden Delicious’ (‘GD’) and ‘Braeburn’ (‘Br’), were analyzed with respect to antioxidants and selected antioxidant enzyme activity after inoculation with *B. cinerea*. The increase in SOD and APX, as well as the decrease in POX in inoculated tissue (Table S4a) are a result of the response in apple during disease, because these changes were only observed in susceptible tissue (Table S4b). The results are summarized in a flow chart (Fig. 3).

As SOD is an important part of plant detoxification of ROS, the increase in SOD activity observed is an attempted plant defense strategy. Superoxide dismutase activity could possibly also originate from *B. cinerea* because *BCSOD1* was found to be a virulence factor for *B. cinerea* in *Phaseolus vulgaris*,^26^ and in *Arabidopsis thaliana* and tomato plants.^27^ However, *B. cinerea* SOD activity has also been described as redundant, because a *bap1* deletion strain of *B. cinerea*, defective in the transcription of several genes for ROS detoxification systems, displayed no change in virulence on bean, *A. thaliana*, apple, or tomato; nor did the absence of antioxidant enzymes affect its ability to survive in infected plants.^28^

Inoculation with *B. cinerea* led to a strong increase in ‘Br’ APX activity in heavily diseased tissue, particularly in the flesh of the shaded side. The increase in APX activity may suggest an attempt by the host to metabolize the H_2_O_2_ generated from other ROS during disease. The increase in APX activity was accompanied by a decrease in total vitamin C levels both in flesh and peel tissues over the course of the experiment. This presumably occurred because disease perturbed the redox equilibrium. Thus, as the disease became more severe, vitamin C was no longer recycled but was being consumed as a consequence of APX activity.

The POX activity was higher in ‘Br’ than in ‘GD’ tissues, in accordance with previous results.^9^ Flavonoid peroxidase activity on the shaded side of ‘Br’ at 14 days was lower in the diseased flesh tissue than in control and mock‐inoculated fruit. The low POX expression correlated well with the low amount of phenolics and progression of disease that was found at 14 days in the susceptible ‘Br’. This could possibly indicate that the availability of the enzyme is regulated by, or dependent on, the availability of its substrate. Alternatively, the POX pathway may have been downregulated by *B. cinerea*, as was previously suggested from inoculation experiments with *B. cinerea* on bean (*Phaseolus vulgaris*) leaf discs.^11^

Plant CAT activity is essential to plant health, particularly during gray mold infection,^12^ but also when plants are not infected. CAT activity remained unchanged in ‘Br’ apples after inoculation and during the course of disease development. Some of the measured activity could have been of *B. cinerea* origin, as seven *CAT* genes are present in its genome and CAT activity has been reported from growth on culture media.^29^ However, it is unlikely, because deletion of one of the catalase genes did not affect virulence^30^ and because of the importance of other detoxification systems – see below.

In theory, the enzyme activities analyzed here have also been detected in *B. cinerea* when grown on culture media.^29^ These are needed for pathogen survival on artificial media supplemented with H_2_O_2_ at levels encountered during infection and disease development.^28^ Some of the antioxidant activity that was measured in the fruit samples could therefore have originated from *B. cinerea*. However, the pathogen does not appear to need all these enzymes when invading a number of different plant species.^14^, ^28^, ^30^ Instead, recent evidence suggests that thioredoxin and thioredoxin reductase have more crucial roles in *B. cinerea* pathogenicity because they enable the pathogen to survive and respond to high levels of oxidative stress.^31^ The elevated antioxidant enzyme activities detected in ‘Br’ during onset of disease proved unsuccessful as a defense strategy to *B. cinerea*.

‘Br’ apple fruit display red, sun‐exposed, and green, shaded sides, respectively. The sun‐exposed side contains several antioxidant pigments that accumulate in the apple peel as a consequence of sun exposure.^32^, ^33^ Common pigments found in apples belong to groups of compounds, such as anthocyanins, flavanols, and carotenoids.^8^ The anthocyanins contribute with red, purple and blue colors.^33^ The flavanols consist mostly of quercetin glycosides that accumulate in the fruit during ripening in the field and are also stably maintained in the fruit after a long storage period in the dark.^34^ Some of these compounds may also have antifungal activity, such as, for instance, quercetin‐3‐galactoside, which inhibits germ tube elongation of *B. cinerea*.^35^

Quercetin is one of the most potent plant antioxidants.^36^ ‘GD’, the less susceptible cultivar, was richer than ‘Br’ in quercetin and its glucosides in both flesh and peel.^1^ These compounds are classified as phenolics, and were found to have been consumed in the diseased apples of ‘Br’, at 14 days post inoculation.

Ascorbic acid is the most abundant, water‐soluble plant antioxidant. It is very important for maintaining redox homeostasis when plants are subjected to environmental stressors that involve ROS.^7^ Thus, it could play a role in plant defense against necrotrophs. Across eight Belgian commercial apple cultivars that had been artificially inoculated with *B. cinerea*, there was a correlation between low AsA levels at harvest and disease development, and between high levels of AsA and less severe disease development.^10^ Similarly, in the present study, a higher accumulation of total vitamin C was detected in the sun‐exposed, less susceptible side than in the shaded side. Total vitamin C levels were also higher in peel than in flesh, which has been observed previously, and this difference persists throughout periods of cold storage.^37^ Total vitamin C levels decreased during the course of disease development in ‘Br’ apples, presumably, because they were being consumed in the decaying tissue while acting as a substrate for APX in the detoxification of H_2_O_2_. In healthy tissue AsA is recycled, and appears to accumulate gradually in response to high light regimes.^38^

‘Br’ was susceptible to *B. cinerea* whereas ‘GD’ was essentially tolerant or even resistant, which has been observed previously.^10^, ^39^ Significant changes in enzyme activity and antioxidant content were found in ‘Br’ over time. In contrast, there was no antioxidant enzyme induction in ‘GD’ and no change in total vitamin C or phenolic content as a result of inoculation. The initial phenolic content was higher in ‘Br’ as compared to ‘GD’ in flesh tissue. Nonetheless, ‘Br’ was more susceptible. Similarly, ‘Br’ displayed higher initial total vitamin C content in peel and higher SOD and POX activities in flesh tissues as compared to ‘GD’. The distinct spectra and quantities of specific phenolic compounds present in the two cultivars may have influenced their susceptibility. But overall, the resistance of ‘GD’ must depend on other factors than those investigated here.

*Botrytis cinerea* produces phytotoxic compounds, such as botrydial and botcinolides, as well as cell‐wall‐degrading enzymes.^40^, ^41^ *B. cinerea* like other postharvest pathogens modulate the host pH environment during infection, and low pH is conducive to gray mold development.^42^, ^43^ *Botrytis cinerea* secretes a number of organic acids, of which citric acid appears to play an important role in its interaction with host plant tissue.^44^ Oxalic acid, another organic acid that is implicated in virulence in *Sclerotinia sclerotiorum*, a close relative to *B. cinerea*, does not appear to be involved in the gray mold‐apple interaction.^43^, ^44^

Based on the current view of the strategy of *B. cinerea* to deal with ROS during pathogenesis, it is reasonable to suggest that the observed increase in antioxidant enzyme activity (SOD and APX) in the susceptible apple tissues were of apple origin. Particularly at the last time point, both host and pathogen would have attempted to maintain cellular redox homeostasis and they would therefore have tried to activate effective antioxidant systems. Future studies could continue to address the nature of the antioxidant enzyme activities, which originate from fruit or fungus, respectively. It would also be desirable to clarify the importance of the timing of the various antioxidant responses that operate during different steps of pathogenesis.

## CONCLUSIONS

The changes detected in antioxidant enzyme activity over time are consistent with the need for ROS detoxification in diseased tissue. The results indicate that high exposure of apple fruit to sun stress in the field might lead to an improved tolerance to postharvest *B. cinerea* infection. Genetic differences in disease tolerance underlie the relative difference in susceptibility between the two cultivars, but the tolerance mechanism does not appear to involve antioxidant metabolism. Further studies are needed to investigate the nature of cultivar differences in their tolerance and susceptibility to postharvest gray mold.

## Supporting information


**Table S1a.** *P*‐values and degrees of freedom from a MANOVA model including tissue as predictor, excluding phenolic content as dependent variable (component). Lesion size was identical for the two levels of tissue (peel and flesh), therefore tissue is not a relevant predictor for the dependent variable lesion size (accordingly, NA in the table).
**Table S1b.** *P*‐values and degrees of freedom from a MANOVA model, excluding tissue as predictor and including phenolic content as dependent variable (component).
**Table S2.** Antioxidant content and antioxidant enzyme activity of peel and flesh tissues of sun‐exposed and shaded sides of apple fruit of the cultivars ‘Braeburn’ and ‘Golden Delicious’, at the start of the experiment (0 days post inoculation).
**Table S3**. Changes over time in total vitamin C content and superoxide dismutase activity in peel tissues for the apple cultivar ‘Braeburn’ on sun‐exposed and shaded sides of fruit.
**Table S4a.** Pairwise comparison of antioxidant enzyme activity for different treatments of the apple cultivars ‘Braeburn’ and ‘Golden Delicious’ at 14 days post inoculation.
**Table S4b.** Pairwise comparison of antioxidant enzyme activity for different treatments and responses in the cultivar ‘Braeburn’ at 14 days post inoculation. Values from all fruit tissue types (sun‐exposed and shaded sides, peel and flesh) are included.

## References

[1] De Paepe D , Valkenborg D , Noten B , Servaes K , Diels L , De Loose M *et al*, Variability of the phenolic profiles in the fruits from old, recent and new apple cultivars cultivated in Belgium. Metabolomics 11:739–752 (2015).

[2] Elad Y , Williamson B , Tudzynski P and Delen N , Chapter 1: *Botrytis spp*. and disease they cause in agricultural systems – an introduction, in Botrytis: Biology, Pathology and Control, ed. by Elad Y, Williamson B, Tudzynski P and Delen N Springer, Dordrecht, The Netherlands, pp. 1–8 (2007).

[3] Romanazzi G and Feliziani E , *Botrytis cinerea* (Gray Mold), in Postharvest Decay Control Strategies, ed. by Bautista‐Banos S Elsevier Inc., London, pp. 131–146 (2014). 10.1016/B978-0-12-411552-1.00004-1.

[4] Sharma P , Jha AB , Dubey RS and Pessarakli M , Reactive oxygen species, oxidative damage, and antioxidative defense mechanism in plants under stressful conditions. J Bot 2012:217037, 26 pages (2012). 10.1155/2012/217037.

[5] Halliwell B , Reactive species and antioxidants. Redox biology is a fundamental theme of aerobic life. Plant Physiol 141:312–322 (2006). 16760481 10.1104/pp.106.077073 PMC1475431

[6] Foyer CH and Noctor G , Review: Ascorbate and glutathione: the heart of the redox hub. Plant Physiol 155:2–18 (2011). 21205630 10.1104/pp.110.167569 PMC3075780

[7] Gallie DR , The role of l‐ascorbic acid recycling in responding to environmental stress and in promoting plant growth. J Exp Bot 64:433–443 (2013). 23162122 10.1093/jxb/ers330

[8] Solovchenko A and Schmitz‐Eiberger M , Significance of skin flavonoids for UV‐B‐protection in apple fruits. J Exp Bot 54:1977–1984 (2003). 12815032 10.1093/jxb/erg199

[9] Zupan A , Mikulic‐Petkovsek M , Slatnar A , Stampar F and Veberic R , Individual phenolic response and peroxidase activity in peel of differently sun‐exposed apples in the period favorable for sunburn occurrence. J Plant Physiol 171:1706–1712 (2014). 25209696 10.1016/j.jplph.2014.08.010

[10] Davey MW , Auwerkerken A and Keulemans J , Relationship of apple vitamin C and antioxidant contents to harvest date and postharvest pathogen infection. J Sci Food Agric 87:802–813 (2007).

[11] von Tiedemann A , Evidence for a primary role of active oxygen species in induction of host cell death during infection of bean leaves with *Botrytis cinerea* . J Physiol Mol Plant Pathol 50:151–160 (1997).

[12] Govrin EM and Levine A , The hypersensitive response facilitates plant infection by the necrotrophic pathogen *Botrytis cinerea* . Curr Biol 10:751–757 (2000). 10898976 10.1016/s0960-9822(00)00560-1

[13] Noctor G , Lelarge‐Trouverie C and Mhamdi A , The metabolomics of oxidative stress. Phytochemistry 112:33–53 (2015). 25306398 10.1016/j.phytochem.2014.09.002

[14] Siegmund U and Viefhues A , Chapter 14: Reactive oxygen species in the Botrytis – host interaction, in Botrytis – the Fungus, the Pathogen and its Management in Agricultural Systems, ed. by Fillinger S and Elad Y Springer International Publishing, Switzerland, pp. 269–289 (2016).

[15] Tenberge KB , Beckedorf M , Hoppe B , Schouten A , Solf M and Von Den Driesch M , *In situ* localization of AOS in host‐pathogen interactions. Microsc Microanal 8:250–251 (2002).

[16] Heller J and Tudzynski P , Reactive oxygen species in phytopathogenic fungi: signaling, development and disease. Annu Rev Phytopathol 49:369–390 (2011). 21568704 10.1146/annurev-phyto-072910-095355

[17] Broekaert WF , Terras FRG , Cammue BPA and Vandedeyde J , An automated quantitative assay for fungal growth inhibition. FEMS Microbiol Lett 69:55–59 (1990).

[18] Ahn T , Paliyath G and Murr DP , Antioxidant enzyme activities in apple varieties and resistance to superficial scald development. Food Res Int 40:1012–1019 (2007).

[19] Bradford MM , A rapid and sensitive method for the quantitation of microgram quantities of protein utilizing the principle of protein‐dye binding. Anal Biochem 72:248–254 (1976). 942051 10.1016/0003-2697(76)90527-3

[20] Banowetz GM , Dierksen KP , Azevedo MD and Stout R , Microplate quantification of plant leaf superoxide dismutases. Anal Biochem 332:314–320 (2004). 15325300 10.1016/j.ab.2004.06.015

[21] Chance B and Maehly AC , Assay of catalases and peroxidases. Methods Enzymol 2:764–775 (1955). 10.1002/9780470110171.ch14 13193536

[22] Nakano Y and Asada K , Hydrogen peroxide is scavenged by ascorbate‐specific peroxidase in spinach chloroplasts. Plant Cell Physiol 22:867–880 (1981).

[23] Aebi H , Catalase in vitro. Methods Enzymol 105:121–126 (1984). 6727660 10.1016/s0076-6879(84)05016-3

[24] Singleton VL , Orthofer R and Lamuela‐Raventos RM , Analysis of total phenols and other oxidation substrates and antioxidants by means of Folin‐Ciocalteu Reagent. Methods Enzymol 299:152–177 (1999).

[25] Franck C , Baetens M , Lammertyn J , Verboven P , Davey MW and Nicolai BM , Ascorbic acid concentration in cv. Conference pears during fruit development and postharvest storage. J Agric Food Chem 51:4757–4763 (2003). 14705909 10.1021/jf026229a

[26] Rolke Y , Liu S , Quidde T , Williamson B , Schouten A , Weltring K‐M *et al*, Functional analysis of H_2_O_2_‐generating systems in *Botrytis cinerea*: the major Cu‐Zn‐superoxide dismutase (BCSOD1) contributes to virulence on French bean, whereas a glucose oxidase (BCGOD1) is dispensable. Mol Plant Pathol 5:17–27 (2004). 20565578 10.1111/j.1364-3703.2004.00201.x

[27] López‐Cruz J , Crespo‐Salvador O , Fernandez‐Crespo E , Garcia‐Agustin P and Gonzalez‐Bosch C , Absence of Cu‐Zn superoxide dismutase BCSOD1 reduces *Botrytis cinerea* virulence in Arabidopsis and tomato plants, revealing interplay among reactive oxygen species, callose and signalling pathways. Mol Plant Pathol 18:16–31 (2017). 26780422 10.1111/mpp.12370 PMC6638242

[28] Temme N and Tudzynski P , Does *Botrytis cinerea* ignore the H_2_O_2_‐induced oxidative stress during infection? Characterization of *Botrytis* Activator Protein 1. Mol Plant Microbe Interact 22:987–998 (2009). 19589074 10.1094/MPMI-22-8-0987

[29] Gil‐ad NL , Bar‐Nun N , Noy T and Mayer AM , Enzymes of *Botrytis cinerea* capable of breaking down hydrogen peroxide. FEMS Microbiol Lett 190:121–126 (2000). 10981701 10.1111/j.1574-6968.2000.tb09273.x

[30] Schouten A , Tenberge KB , Vermeer J , Stewart J , Wagemakers L , Williamson B *et al*, Functional analysis of an extracellular catalase of *Botrytis cinerea* . Mol Plant Pathol 3:227–238 (2002). 20569330 10.1046/j.1364-3703.2002.00114.x

[31] Viefhues A , Heller J , Temme N and Tudzynski P , Redox systems in *Botrytis cinerea*: impact on development and virulence. Mol Plant Microbe Interact 27:858–874 (2014). 24983673 10.1094/MPMI-01-14-0012-R

[32] Li P , Ma F and Cheng L , Primary and secondary metabolism in the sun‐exposed peel and the shaded peel of apple fruit. Physiol Plant 148:9–24 (2013). 22989296 10.1111/j.1399-3054.2012.01692.x

[33] Sharma M , Sitbon C , Subramanian J and Paliyath G , Chapter 21: Changes in nutritional quality of fruits and vegetables during storage, in Postharvest Biology and Technology of Fruits, Vegetables, and Flowers, ed. by Paliyath G, Murr DP, Handa AK and Lurie S Wiley‐Blackwell Publishing, Ames, Iowa, USA, pp. 444–465 (2008).

[34] Awad MA and De Jager A , Flavonoid and chlorogenic acid concentrations in skin of ‘Jonagold’ and ‘Elstar’ apples during and after regular and ultra‐low oxygen storage. Postharvest Biol Biotechnol 20:15–24 (2000).

[35] Tao S , Zhang S , Tsao R , Charles MT , Yang R and Khanizadeh S , *In vitro* antifungal activity and mode of action of selected polyphenolic antioxidants on *Botrytis cinerea* . Arch Phytopathol Pflanzenschutz 43:1564–1578 (2010).

[36] Rice‐Evans CA , Miller NJ and Paganga G , Structure‐antioxidant activity relationships of flavonoids and phenolic acids. Free Radic Biol Med 20:933–956 (1996). 8743980 10.1016/0891-5849(95)02227-9

[37] Felicetti E and Mattheis JP , Quantification and histochemical localization of ascorbic acid in ‘delicious,’ ‘Golden delicious,’ and ‘Fuji’ apple fruit during on‐tree development and cold storage. Postharvest Biol Biotechnol 56:56–63 (2010).

[38] Davey MW , Van Montagu M , Inze D , Sanmartin M , Kanellis A , Smirnoff N *et al*, Plant L‐ascorbic acid: chemistry, function, metabolism, bioavailability and effects of processing. J Sci Food Agric 80:825–860 (2000).

[39] Spotts RA , Cervantes LA and Mielke EA , Variability in postharvest decay among apple cultivars. Plant Dis 83:1051–1054 (1999). 30841275 10.1094/PDIS.1999.83.11.1051

[40] van Kan JAL , Review: licensed to kill: the lifestyle of a necrotrophic plant pathogen. Trends Plant Sci 11:247–253 (2006). 16616579 10.1016/j.tplants.2006.03.005

[41] Williamson B , Tudzynski B , Tudzynski P and van Kan JAL , *Botrytis cinerea*: the cause of grey mould disease. Mol Plant Pathol 8:561–580 (2007). 20507522 10.1111/j.1364-3703.2007.00417.x

[42] Liu J , Sui Y , Wisniewski M , Xie Z , Liu Y , You Y *et al*, The impact of the postharvest environment on the viability and virulence of decay fungi. Crit Rev Food Sci 58:1681–1687 (2018). 10.1080/10408398.2017.1279122 28140651

[43] Rascle C , Dieryckx C , Dupuy JW , Muszkieta L , Souibgui E , Droux M *et al*, The pH regulator PacC: a host‐dependent virulence factor in *Botrytis cinerea* . Environ Microbiol Rep 10:555–568 (2018). 30066486 10.1111/1758-2229.12663

[44] Müller N , Leroch M , Schumacher J , Zimmer D , Könnel A , Klug K *et al*, Investigations on VELVET regulatory mutants confirm the role of host tissue acidification and secretion of proteins in the pathogenesis of *Botrytis cinerea* . New Phytol 219:1062–1074 (2018). 29790574 10.1111/nph.15221

