# Supplementary material for: Botrytis cinerea differentially induces postharvest antioxidant responses in ‘Braeburn’ and ‘Golden Delicious’ apple fruit
Source: J Sci Food Agric. 2019 Jul 8;99(13):5662–70. doi: 10.1002/jsfa.9827 (PMC6771965; doi:10.1002/jsfa.9827)
Supplement: Supplementary file 1 — Table S1a. P‐values and degrees of freedom from a MANOVA model including tissue as predictor, excluding phenolic content as dependent variable (component). Lesion size was identical for the two levels of tissue (peel and flesh), therefore tissue is not a relevant predictor for the dependent variable lesion size (accordingly, NA in the table). Table S1b. P‐values and degrees of freedom from a MANOVA model, excluding tissue as predictor and including phenolic content as dependent variable (component). Table S2. Antioxidant content and antioxidant enzyme activity of peel and flesh tissues of sun‐exposed and shaded sides of apple fruit of the cultivars ‘Braeburn’ and ‘Golden Delicious’, at the start of the experiment (0 days post inoculation). Table S3. Changes over time in total vitamin C content and superoxide dismutase activity in peel tissues for the apple cultivar ‘Braeburn’ on sun‐exposed and shaded sides of fruit. Table S4a. Pairwise comparison of antioxidant enzyme activity for different treatments of the apple cultivars ‘Braeburn’ and ‘Golden Delicious’ at 14 days post inoculation. Table S4b. Pairwise comparison of antioxidant enzyme activity for different treatments and responses in the cultivar ‘Braeburn’ at 14 days post inoculation. Values from all fruit tissue types (sun‐exposed and shaded sides, peel and flesh) are included. [file JSFA-99-5662-s001.docx]

| **Table S1a.** *P*-values and degrees of freedom from a MANOVA model including tissue as predictor, excluding phenolic content as dependent variable (component). Lesion size was identical for the two levels of tissue (peel and flesh), therefore tissue is not a relevant predictor for the dependent variable lesion size (accordingly, NA in the table). | | | | | | | | | | | | | | | | |
| --- | --- | --- | --- | --- | --- | --- | --- | --- | --- | --- | --- | --- | --- | --- | --- | --- |
|  | | **Source of Variation** | | | | | **Interactions** | | | | | | | | | |
|  |  | Treatment (A) | Cultivar (B) | Side (C) | Tissue (D) | Day (E) | AB | AC | AD | AE | BC | BD | BE | CD | CE | DE |
| **df^†^** | | 2 | 1 | 1 | 1 | 2 | 2 | 2 | 2 | 2 | 1 | 1 | 2 | 1 | 2 | 2 |
| **Overall MANOVA** | | 10^-16^ | 10^-16^ | 10^-15^ | 10^-16^ | 10^-16^ | 10^-13^ | NS | 10^-10^ | 10^-13^ | 10^-5^ | 10^-10^ | 10^-5^ | 10^-10^ | NS | 10^-15^ |
| **Components**^†^ | |  |  |  |  |  |  |  |  |  |  |  |  |  |  |  |
|  | Lesion  size | 10^-16^ | 10^-9^ | 0.01 | NA | 10^-11^ | 10^-15^ | 0.02 | NA | 10^-15^ | NS | NA | 10^-7^ | NA | 0.04 | NA |
|  | Vit C | 10^-5^ | 10^-16^ | 10^-16^ | 10^-16^ | 0.03 | 10^-6^ | NS | 0.04 | NS | 10^-7^ | 10^-11^ | NS | 10^-12^ | NS | 10^-4^ |
|  | Phe | - | - | - | - | - | - | - | - | - | - | - | - | - | - | - |
|  | SOD | 10^-8^ | 10^-4^ | NS | 10^-16^ | 0.04 | 10^-3^ | NS | 10^-10^ | 10^-6^ | NS | NS | NS | NS | NS | 10^-7^ |
|  | POX | 0.03 | 10^-12^ | NS | 10^-16^ | 10^-3^ | NS | NS | NS | NS | NS | NS | NS | NS | NS | 10^-8^ |
|  | APX | 10^-3^ | NS | NS | 10^-8^ | NS | NS | NS | 10^-3^ | NS | NS | NS | NS | NS | NS | 0.04 |
|  | CAT | NS | NS | NS | 10^-13^ | 10^-13^ | NS | NS | NS | NS | NS | NS | NS | NS | NS | 10^-3^ |
| ^†^Abbreviations: df, degrees of freedom; Vit C, total vitamin C content; Phe, phenolic content; SOD, superoxide dismutase activity, POX, flavonoid peroxidase activity; APX, ascorbate peroxidase activity; CAT, catalase activity; NA, not applicable; NS, no significant difference (*P* > 0.05). | | | | | | | | | | | | | | | | |

| **Table S1b.** *P*-values and degrees of freedom from a MANOVA model, excluding tissue as predictor and including phenolic content as dependent variable (component). | | | | | | | | | | | | | | | | |
| --- | --- | --- | --- | --- | --- | --- | --- | --- | --- | --- | --- | --- | --- | --- | --- | --- |
|  | | **Source of Variation** | | | | | **Interactions** | | | | | | | | | |
|  |  | Treatment (A) | Cultivar (B) | Side (C) | Tissue (D) | Day (E) | AB | AC | AD | AE | BC | BD | BE | CD | CE | DE |
| **df^†^** | | 2 | 1 | 1 | - | 2 | 2 | 2 | - | 2 | 1 | - | 2 | - | 2 | - |
| **Overall MANOVA** | | 10^-11^ | 10^-16^ | 0.08 | - | 10^-16^ | 10^-9^ | NS | - | 10^-4^ | NS | - | 10^-10^ | - | NS | - |
| **Components**^†^ | |  |  |  |  |  |  |  |  |  |  |  |  |  |  |  |
|  | Lesion size | 10^-12^ | 10^-5^ | 0.07 | - | 10^-6^ | 10^-8^ | NS | - | 10^-7^ | NS | - | 10^-4^ | - | NS | - |
|  | Vit C | 10^-3^ | 10^-11^ | 0.01 | - | 0.01 | 0.06 | NS | - | NS | 0.05 | - | 10^-3^ | - | NS | - |
|  | Phe | NS | NS | NS | - | 10^-3^ | 0.01 | NS | - | NS | NS | - | NS | - | 0.05 | - |
|  | SOD | 10^-6^ | 10^-16^ | NS | - | 10^-15^ | 10^-3^ | NS | - | NS | NS | - | 10^-8^ | - | NS | - |
|  | POX | 0.06 | 10^-5^ | NS | - | 10^-5^ | NS | NS | - | NS | NS | - | 0.02 | - | NS | - |
|  | APX | NS | NS | NS | - | NS | 10^-3^ | NS | - | 10^-4^ | NS | - | 10^-3^ | - | NS | - |
|  | CAT | NS | NS | NS | - | 10^-8^ | 0.05 | NS | - | NS | NS | - | NS | - | 0.06 | - |
| ^†^Abbreviations: df, degrees of freedom; Vit C, total vitamin C content; Phe, phenolic content; SOD, superoxide dismutase activity, POX, flavonoid peroxidase activity; APX, ascorbate peroxidase activity; CAT, catalase activity; NS, no significant difference (*P* > 0.05). | | | | | | | | | | | | | | | | |

| **Table S2.** Antioxidant content and antioxidant enzyme activity of peel and flesh tissues of sun-exposed and shaded sides of apple fruit of the cultivars ‘Braeburn’ and ‘Golden Delicious’, at the start of the experiment (0 d post inoculation). | | | | | | | | | |
| --- | --- | --- | --- | --- | --- | --- | --- | --- | --- |
| **Type of sample** | | | **Vit C^†^**  **(g kg^-1^ FW^†^)** | | **Phe**^†^  **(mg GAE^†^ kg^-1^ FW)** | **SOD^†^**  **(units g^-1^ FW)** | **POX^†^**  **(units g^-1^ FW)** | **APX^†^**  **(units g^-1^ FW)** | **CAT^†^**  **(units g^-1^ FW)** |
| **Cv^†^** | **Tissue** | **Side** | |  |  |  |  |  |  |
| ‘Br’^†^ | Peel | Sun.exp.^†^ | | 0.80 ± 0.17^a^ | NA^†^ | 3.62 ± 2.31^ab^ | 0.79 ± 0.41^c^ | 0.08 ± 0.05^a^ | 0.18 ± 0.04^ac^ |
|  |  | Shaded | | 0.18 ± 0.12^b^ | NA | 2.93 ± 1.28^ac^ | 0.71 ± 0.23^c^ | 0.06 ± 0.04^a^ | 0.10 ± 0.04^bc^ |
|  | Flesh | Sun.exp. | | 0.08 ± 0.03^b^ | 1.01 ± 0.11^a^ | 4.73 ± 1.17^a^ | 4.80 ± 1.07^a^ | 0.05 ± 0.04^a^ | 0.45 ± 0.24^ab^ |
|  |  | Shaded | | 0.05 ± 0.02^b^ | 0.83 ± 0.17^ab^ | 3.75 ± 1.45^ab^ | 5.35 ± 0.52^a^ | 0.03 ± 0.02^a^ | 0.51 ± 0.29^a^ |
| ‘GD’^†^ | Peel | Sun.exp. | | 0.14 ± 0.10^b^ | NA | 1.43 ± 0.90^bc^ | 0.33 ± 0.16^c^ | 0.05 ± 0.04^a^ | 0.06 ± 0.04^c^ |
|  |  | Shaded | | 0.05 ± 0.04^b^ | NA | 1.77 ± 0.64^bc^ | 0.15 ± 0.09^c^ | 0.03 ± 0.03^a^ | 0.02 ± 0.02^c^ |
|  | Flesh | Sun.exp. | | 0.01 ± 0.02^b^ | 0.67 ± 0.1^b^ | 0.76 ± 1.10^c^ | 3.36 ± 0.80^b^ | 0.03 ± 0.02^a^ | 0.26 ± 0.22^ac^ |
|  |  | Shaded | | 0.01 ± 0.01^b^ | 0.71 ± 0.11^b^ | 0.71 ± 1.09^c^ | 3.15 ± 0.97^b^ | 0.03 ± 0.03^a^ | 0.32 ± 0.30^ac^ |
| ^†^Abbreviations: Cv, Cultivar; ‘Br’, ‘Braeburn’; ‘GD’, ‘Golden Delicious’; Sun.exp., sun-exposed; Vit C, total vitamin C content (AsA + DHA); AsA, ascorbic acid; DHA, dehydroascorbate; FW, fresh weight; Phe, phenolic content; GAE, gallic acid equivalents; SOD, superoxide dismutase activity, POX, flavonoid peroxidase activity; APX, ascorbate peroxidase activity; CAT, catalase activity; NA: Not applicable (no skin/peel tissue available).  Data were analyzed by ANOVA, followed by Tukey’s test. Data are presented as means ± SD of ten replicates. Values not sharing the same letters reading down are significantly different at *P* < 0.05. | | | | | | | | | |

| **Table S3**. Changes over time in total vitamin C content and superoxide dismutase activity in peel tissues for the apple cultivar ‘Braeburn’ on sun-exposed and shaded sides of fruit. | | | | | |
| --- | --- | --- | --- | --- | --- |
| **Treatments**^†^ | **dpi**^††^ | **Vit C**^††^ **(g kg^-1^ FW^†^**^†^**)** | | **SOD**^††^ **(units g^-1^ FW)** | |
|  |  | Sun-exposed | Shaded | Sun-exposed | Shaded |
| Control | 0 | 0.80 ± 0.23^a^ | 0.18 ± 0.17^de^ | 3.62 ± 3.22^b^ | 2.93 ± 1.78^b^ |
| Control | 5 | 0.68 ± 0.38^abc^ | 0.44 ± 0.15^abcde^ | 4.92 ± 3.11^b^ | 6.35 ± 3.42^b^ |
| Mock | 5 | 0.73 ± 0.09^ab^ | 0.38 ± 0.18^bcde^ | 5.00 ± 3.02^b^ | 7.29 ± 3.66^b^ |
| Inoculated | 5 | 0.56 ± 0.31^abcd^ | 0.16 ± 0.13^de^ | 4.33 ± 3.16^b^ | 7.00 ± 4.91^b^ |
| Control | 14 | 0.57 ± 0.21^abcd^ | 0.28 ± 0.15^bcde^ | 7.13 ± 7.80^b^ | 6.46 ± 5.84^b^ |
| Mock | 14 | 0.64 ± 0.26^abc^ | 0.31 ± 0.28^bcde^ | 2.50 ± 1.49^b^ | 3.56 ± 2.34^b^ |
| Inoculated | 14 | 0.23 ± 0.38^cde^ | 0.00 ± 0.00^e^ | 25.79 ± 23.16^a^ | 23.38 ± 10.99^a^ |
| ^†^Treatments: Control (neither wounded nor inoculated), Mock-inoculated (wounded and treated with sterile distilled water) and *B. cinerea*-inoculated (wounded and inoculated with 10 µL of a 1.5 x 10^5^ mL^-1^ *B. cinerea* spore suspension).  ^††^Abbreviations: dpi, d post inoculation; Vit C, total vitamin C content (AsA + DHA); AsA, ascorbic acid; DHA, dehydroascorbate; FW, fresh weight; SOD, superoxide dismutase activity.  Pair-wise comparisons of means was done with R function emmeans, combined with Tukey’s test. The family-wise error rate was controlled at a predetermined alfa=0.05. Values not sharing the same letters, reading down, within total vitamin C content and SOD columns, respectively, are significantly different at *P* < 0.05. Values are means ± SD of six to ten replicates. | | | | | |

| **Table S4a.** Pairwise comparison of antioxidant enzyme activity for different treatments of the apple cultivars ‘Braeburn’ and ‘Golden Delicious’ at 14 d post inoculation. | | | | | | | | |
| --- | --- | --- | --- | --- | --- | --- | --- | --- |
| **Cv^†^** | **Enzyme^†^** | **Enzyme activity**  **(units g^-1^ FW^†^)** | | | **Pairwise t-tests^††^** | | | **Inoculated compared to control** |
|  |  | Control | Mock | Inoculated | Control vs. Mock | Mock vs. Inoculated | Control vs. Inoculated | Increase or decrease (+ or -) |
| ‘Br’**^†^** | SOD | 3.48 ± 5.67 | 1.63 ± 2.00 | 12.58 ± 17.19 | 0.14 | 0.003^**^ | **0.018^*^** | **+ 3.6-fold** |
|  | POX | 2.16 ± 1.09 | 2.63 ± 1.87 | 1.25 ± 1.16 | 0.29 | 0.0037^**^ | **0.008^**^** | **- 1.7-fold** |
|  | APX | 0.04 ± 0.04 | 0.16 ± 0.29 | 0.10 ± 0.12 | 0.051 | 0.37 | **0.019^*^** | **+ 2.5-fold** |
|  | CAT | 1.28 ± 1.54 | 0.88 ± 1.09 | 0.63 ± 0.78 | 0.30 | 0.037^*^ | 0.072 | NS^†^ |
| ‘GD’**^†^** | SOD | 2.78 ± 3.65 | 2.93 ± 3.70 | 4.76 ± 5.57 | 0.88 | 0.19 | 0.15 | NS |
|  | POX | 1.25 ± 0.97 | 1.46 ± 1.44 | 1.41 ± 1.28 | 0.56 | 0.89 | 0.64 | NS |
|  | APX | 0.06 ± 0.07 | 0.03 ± 0.04 | 0.09 ± 0.11 | 0.13 | 0.016^*^ | 0.21 | NS |
|  | CAT | 0.91 ± 0.76 | 0.73 ± 1.04 | 0.56 ± 0.72 | 0.51 | 0.50 | 0.11 | NS |
| ^†^Abbreviations: Cv, Cultivar; ‘Br’, ‘Braeburn’; ‘GD’, ‘Golden Delicious’; SOD, superoxide dismutase activity, POX, flavonoid peroxidase activity; APX, ascorbate peroxidase activity; CAT, catalase activity; FW, fresh weight; NS, no significant difference (*P* > 0.05).  ^††^The *P*-values resulting from an ANOVA followed by pairwise t-tests with non-pooled standard deviation and no correction for multiple comparisons for the three treatments: control, mock-inoculated and *B. cinerea*-inoculated are presented. Significantly different enzyme activities of control tissue compared to *B. cinerea*-inoculated tissue, with comparisons of means translated into fold change, are shown in bold.  *Significantly different at *P* < 0.05; **Significantly different at *P* < 0.01. | | | | | | | | |

| **Table S4b.** Pairwise comparison of antioxidant enzyme activity for different treatments and responses in the cultivar ‘Braeburn’ at 14 d post inoculation. Values from all fruit tissue types (sun-exposed and shaded sides, peel and flesh) are included. | | | | | | | | |
| --- | --- | --- | --- | --- | --- | --- | --- | --- |
| **Enzyme^†^** | **Response^††^** | **Enzyme activity**  **(units g^-1^ FW^†^)** | | | **Pairwise t-tests^†††^** | | | **Inoculated compared to control** |
|  |  | Control | Mock | Inoculated | Control vs. Mock | Mock vs. Inoculated | Control vs. Inoculated | Increase or  decrease (+ or -) |
| SOD |  | 3.48 ± 5.67 | 1.63 ± 2.00 | 12.58 ± 17.19 | 0.14 | 0.003 ^**^ | **0.018 ^*^** | **+ 3.6-fold** |
|  | Tolerant |  |  | 1.38 ± 2.06 |  | 0.84 | 0.20 | NS |
|  | Susceptible |  |  | 14.19 ± 17.60 |  | 0.0077 ^**^ | **0.022 ^*^** | **+ 4.1-fold** |
| POX |  | 2.16 ± 1.09 | 2.63 ± 1.87 | 1.25 ± 1.16 | 0.29 | 0.0037 ^**^ | **0.008 ^**^** | **- 1.7-fold** |
|  | Tolerant |  |  | 1.83 ± 0.56 |  | 0.11 | 0.39 | NS |
|  | Susceptible |  |  | 1.10 ± 1.20 |  | 0.0026 ^**^ | **0.0058 ^**^** | **- 2.0-fold** |
| APX |  | 0.04 ± 0.04 | 0.16 ± 0.29 | 0.10 ± 0.12 | 0.051^*^ | 0.37 | **0.019 ^*^** | **+ 2.5-fold** |
|  | Tolerant |  |  | 0.04 ± 0.06 |  | 0.078 | 0.99 | NS |
|  | Susceptible |  |  | 0.11 ± 0.13 |  | 0.49 | **0.033 ^*^** | **+ 2.8-fold** |
| CAT |  | 1.28 ± 1.54 | 0.88 ± 1.09 | 0.63 ± 0.78 | 0.30 | 0.037^*^ | 0.072 | NS |
|  | Tolerant |  |  | 0.30 ± 0.27 |  | 0.037 ^*^ | **0.008 ^**^** | **- 4.3-fold** |
|  | Susceptible |  |  | 0.68 ± 0.88 |  | 0.51 | 0.12 | NS |
| ^†^Abbreviations: SOD, superoxide dismutase activity, POX, flavonoid peroxidase activity; APX, ascorbate peroxidase activity; CAT, catalase activity; FW, fresh weight; NS, no significant difference (*P* > 0.05).  ^††^ The three treatments were: control, mock-inoculated and *B. cinerea*-inoculated. The latter were divided further, according to their responses, as susceptible (diseased) or tolerant (healthy).  ^††^**^†^**The *P*-values resulting from an ANOVA followed by pairwise t-tests with non-pooled standard deviation and no correction for multiple comparisons are presented. Significant changes in enzyme activities (control vs. inoculated) were calculated as fold changes and transferred into a flow chart (Fig. 3).  *Significantly different at *P* < 0.05; **Significantly different at *P* < 0.01. | | | | | | | | |
